# Reduced miR-29a-3p expression is linked to the cell proliferation and cell migration in gastric cancer

**DOI:** 10.1186/s12957-015-0513-x

**Published:** 2015-03-12

**Authors:** Zhujiang Zhao, Ling Wang, Wei Song, He Cui, Gang Chen, Fengchang Qiao, Jiaojiao Hu, Rongping Zhou, Hong Fan

**Affiliations:** Department of Genetics & Developmental Biology, the Medical School of Southeast University and Key Laboratory of Developmental Genes and Human Diseases, Ministry of Education, Southeast University, 2 Sipailou, Xuanwu, Nanjing, 210009 China; Jiangning Hospital, Nanjing Medical University, 140 Hanzhong Road, Nanjing, 210000 China

**Keywords:** miR-29a-3p, Migration, Cell proliferation, Gastric cancer

## Abstract

**Background:**

MicroRNAs (miRNAs) play an important role in a tumor-suppressive or oncogenic manner in carcinogenesis. Alteration expression patterns of miRNAs in gastric cancer (GC) are associated with cancer initiation and progression. In the present study, we evaluated miR-29a-3p expression pattern and its function in gastric carcinogenesis.

**Methods:**

The expression of miR-29a-3p in GC tissue samples and cell lines was detected by quantitative real-time PCR (qRT-PCR). After transfected with miR-29a-3p mimics or inhibitor, the cell proliferation, cell migration, and invasion ability were assessed by CCK-8 assay, wound healing assay, and Trans-well assay, respectively. The level of CDK2, CDK4, CDK6, and CyclinD1 were determined by qRT-PCR and Western blot.

**Results:**

Compared with the corresponding non-tumor tissues, miR-29a-3p showed a significant down-regulated expression in tumor tissues. *In vitro* functional assays demonstrated that enforced miR-29a-3p expression inhibited cell proliferation by reducing the expression of CDK2, CDK4, and CDK6. Wound healing and Transwell assays revealed that miR-29a-3p suppressed tumor metastasis in GC.

**Conclusions:**

Our preliminary results suggest that altered expression of miR-29a-3p is involved in gastric cancer process. The present study provides the first insight into the specific role of miR-29a-3p in gastric carcinogenesis.

**Electronic supplementary material:**

The online version of this article (doi:10.1186/s12957-015-0513-x) contains supplementary material, which is available to authorized users.

## Background

Gastric cancer (GC) is one of the malignant digestive tract tumors which seriously threatens human health [1], accounting for a major cause of cancer-related mortality in China [2]. Although the tremendous improvements in diagnosis and treatment technologies, the difficulty for early diagnosis and the poor prognosis of advanced GC still results in a low survival rate [3]. Multiple encoding or non-coding gene expression alteration is involved in the occurrence and progression of GC. MicroRNAs (miRNAs) are small endogenous, non-coding, single-stranded RNAs [4], which inhibit the translation and stability of messenger RNAs (mRNAs), controlling genes expression involved in cellular processes such as cell-cycle regulation, differentiation, apoptosis, and migration. Nowadays, it is shown that miRNA closely relates to the process of tumor and the development of tumor invasion and migration [5]. miRNA also acts as a biomarker stably expressed in serum and provides new target for molecular target therapy of various cancers [6]. Accumulating evidences have strongly suggested that altered miRNA expression profiles and patterns could play an important role in tumorigenesis as oncogenes and tumor-suppressor genes [7]. Currently, multiple abnormal expressions of miRNAs in gastric cancer have been observed, which are involved in gastric cancer cell proliferation, invasion, metastasis, and apoptosis, as well as in radiotherapy and chemotherapy sensitivity *via* regulating different tumor-related target genes [8-11]. Hence, the extensive analysis of miRNA expression in GC could contribute to deeply understand the mechanisms of GC development and identify diagnostic biomarkers and therapeutic targets.

miR-29a-3p is a member of miR-29s family, which is a conserved family of miRNA. Decreased expression of miR-29s has been described in multiple cancers, including GC [12,13]. Despite plenty of evidences showing that miR-29s can function as a tumor-suppressor gene, miR-29a-3p expression pattern in GCs still remains partially unresolved. The present study revealed that miR-29a-3p was significantly decreased in GC tissues and was involved in cell growth, migration, and invasion. These studies also provided novel insight into the role of miR-29a-3p in gastric carcinogenesis which would be crucial for the development of new strategies for GC treatment.

## Methods

### Cell culture and oligonucleotides transfection

Human gastric adenoma cell lines, including GES-1, SGC-7901, AGS, MCG803, and BGC-823, were obtained from the Cell Bank of Chinese Academy of Science and maintained in RPMI-1640 medium (Life Technologies, Carlsbad, CA, USA) supplemented with 10% fetal bovine serum (FBS, Invitrogen, Carlsbad, CA, USA), 100 U/ml of penicillin and 100 mg/ml streptomycin (Invitrogen, Carlsbad, CA) in a humidified incubator with 5% CO_2_ atmosphere at 37°C.

miR-29a-3p mimics/inhibitors and negative control molecules (scramble control mimic and inhibitor) were synthesized and purified by the GenePharma Company (Shanghai, China). The sequences were shown in Additional file 1: Table S1. They were transfected into cells at a final concentration of 50 nM using Lipofectamine-2000 transfection reagent (Invitrogen, Carlsbad, CA, USA) according to the manufacturer’s protocol.

### Tissue sample preparation

Fifty pairs of histopathologically confirmed GC tissues and their adjacent non-cancerous tissue specimens were collected between 2010 and 2013 from the Jiangning Hospital of Nanjing, China. The study was approved by the Committee for Ethical Review of Research at the Jiangning Hospital of Nanjing in China, and the patients signed informed consent forms. All tissue samples were obtained from patients undergoing GC and immediately snap frozen in liquid nitrogen until RNA and protein extraction.

### Real-time reverse transcriptase quantitative PCR

Total RNA was extracted from cell and tissue samples with Trizol reagent (Invitrogen, Carlsbad, CA). For the detection of miRNA expression, the primers used for stem-loop reverse transcription PCR (RT-PCR) and quantitative PCR (qPCR) were synthesized and purified by RiboBio (Guangzhou, China). The PCR conditions were 95°C for 30 s, followed by 40 cycles of 95°C for 30 s, 60°C for 30 s, and 72°C for 30 s. The reactions were monitored using a preheated real-time instrument (ABI step one, Life Technologies). The relative expression ratio of miRNA in gastric cancer tissues and cells was quantified by the 2^−ΔΔCT^ method. The primer sequences of gene *ITGA6* and *LAMA2* were shown in Additional file 2: Table S2.

### Western blot

Western blots were performed using Mouse anti-CDK2, anti-CDK4, anti-CDK6, and anti-cyclin D1 were purchased as part of the Cell Cycle Regulation Sampler Kit (Cell Signaling Technology, Danvers, MS, USA). Mouse anti-β-actin was purchased from Sigma-Aldrich (St. Louis, MO, USA). Protein detection was performed with Super Signal chemiluminescence substrate (Pierce, Rockford, IL, USA).

### Cell proliferation assay

The miRNA mimics/inhibitor was synthesized and purified by GenePharma Company (Shanghai, China) and transfected into the cells at a final concentration of 50 nM/100 nM using Lipofectamine-2000 transfection reagent (Invitrogen). One day before transfection, 5.0 × 10^3^ BGC-823 cells and MCG803 in a 100-μl growth medium were plated in each well of a 96-well plate. The cells were then transfected with 50 nM of various synthetic miRNA mimics and corresponding control, and 100-nM inhibitor and corresponding control, using Lipofectamine-2000 (Invitrogen) according to the manufacturer’s instruction. A cell counting kit-8 (CCK-8) (Dojindo Laboratories, Kumamoto, Japan) was used to measure the cellular growth following the manufacturer’s instruction. Cell proliferation was assessed at different time points (0, 24, 48, and 72 h). Triplicate independent experiments were performed.

### Migration and invasion assay

Cell mobility was subjected to the wound healing assay analysis as described previously [14]. A scratch wound was generated using a 200-μl pipette tip on confluent cell monolayers in a six-well plate. Cells were then washed with a fresh medium to remove floating cells, and the spread of the wound closure was observed after 48 h and photographed under a microscope (Olympus, Tokyo, Japan). The potential for migration and invasion of transfected cells were evaluated by a Transwell assay (Life Technologies). Cells were grown to 70% confluence and transfected for 24 h with miR-29a-3p mimics or control mimics and miR-29a-3p inhibitor or control inhibitor, respectively. In the migration assay, cells were cultured in a 200-ml medium with 1% FBS in the upper chamber of a non-coated Transwell insert. In the lower chamber, a 600-ml medium with 10% FBS was used as a chemo-attractant to encourage cell migration. In the invasion assay, the upper chamber of the Transwell inserts were coated with 50 ml of 1.0 mg/ml Matrigel (Millipore, Billerica, MA, USA), and cells were plated in the upper chamber of the Matrigel-coated Transwell insert (Millipore, Billerica, MA, USA). After incubated for 24 h, the non-migrating or non-invading cells were gently removed with a cotton swab. All cells were stained using 0.1% crystal violet staining and counted in five fields under an inverted microscope. The independent experiments were repeated three times.

### Statistical analysis

The independent Student’s *t*-test was used to compare the results expressed as mean ± SD between any two pre-selected groups. A *P* value less than 0.05 was considered statistically significant. To determine correlations between variables, Pearson’s correlation coefficient was calculated.

## Results

### miR-29a-3p was decreased in GC tissue samples and cell lines

**Figure 1:**
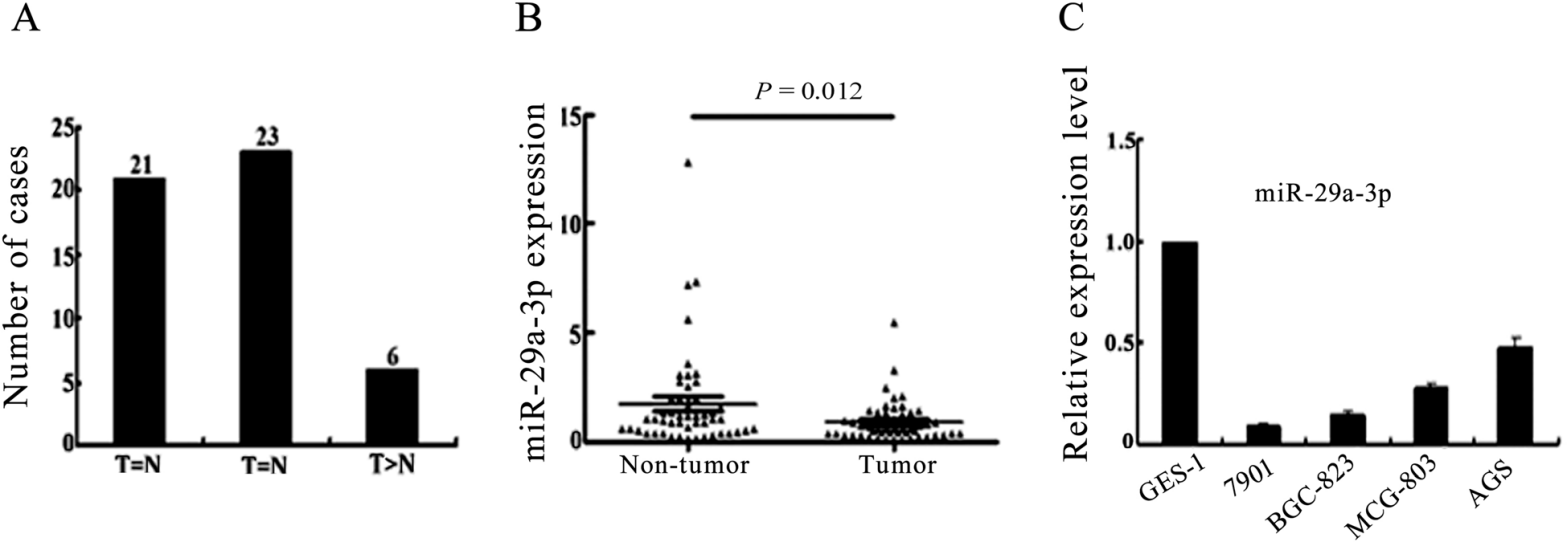
**Decreased expression of miR-29a-3p in GC specimens and cell lines. (A)** qRT-PCR analysis showing miR-29a-3p mRNA level in GC tissues (*n* = 50). The value (defined as “fold difference”) indicated the ratio of the miR-29a-3p mRNA expression level (fold change) in GC tissues to the matched non-tumor tissues. Twenty- one out of fifty GC cases showed a decreased expression of miR-29a-3p (*T* < *N*). The bar chart showed the number of detected GC cases. **(B)** Scatter plots of miR-29a-3p fold change in GC tissues and their matched non-tumor tissues. In both panels, the lines indicated the mean ± SD. The level of miR-29a-3p mRNA was significantly lower in GC tumor tissues compared to the non-tumor counterparts (*P* = 0.012, Paired Student’s *t*-test). **(C)** Semi-quantification of miR-29a-3p expression (fold change) in GC cell lines by qRT-PCR. Data were presented as means ± SD from at least three separate experiments.

To validate the expression of miR-29a-3p in GC tissue samples and cell lines, we conducted quantitative real-time PCR (qRT-PCR) in 50 GC tissues and the corresponding normal tissues and 4 GC cell lines and 1 gastric epithelial cell line (real-time quantitative PCR method in Additional file 3). Compared to the paired non-tumor tissues, 42% (21/50) of GC cases showed decreased expression of miR-29a-3p (defined as greater than a two-fold decrease) (Figure 1A). The average fold change of miR-29a-3p was significantly lower in tumor tissues than that in non-tumor tissues (*P* = 0.012, Paired student’s *t*-test; Figure 1B). Decreased miR-29a-3p was correlated with the degree of cell differentiation (Table 1). Four GC cell lines (SGC-7901, AGS, MCG803, and BGC-823) showed lower level expression of miR-29a-3p compared with the GES-1, normal gastric mucosa cells (Figure 1C). These data indicated that reduced expression of miR-29a-3p might play an important role in gastric carcinogenesis.

**Table 1:** **Correlation of the expression of miR-29a-3p with clinicopathologic feature**

**Clinicopathologic features**	***T*** **≥** ***N***	***T*** **<** ***N***	***P*** **value**
Gender			1
Male	12	19	
Female	5	7	
Differentiation			0.031*
Low	12	9	
Moderate	5	17	
Lymphatic metastasis			0.481
Yes	12	21	
No	5	5	
Infiltration degree			0.071
Tunica mucosa	4	2	
Mucous layer outside	13	17	

Note: clinicopathologic features of 7 GC tissue samples are not included due to imperfection, so only 43 GC tissue samples were analyzed. *Significant differences are shown.

### miR-29a-3p has cell proliferation suppression effects *in vitro*

**Figure 2:**
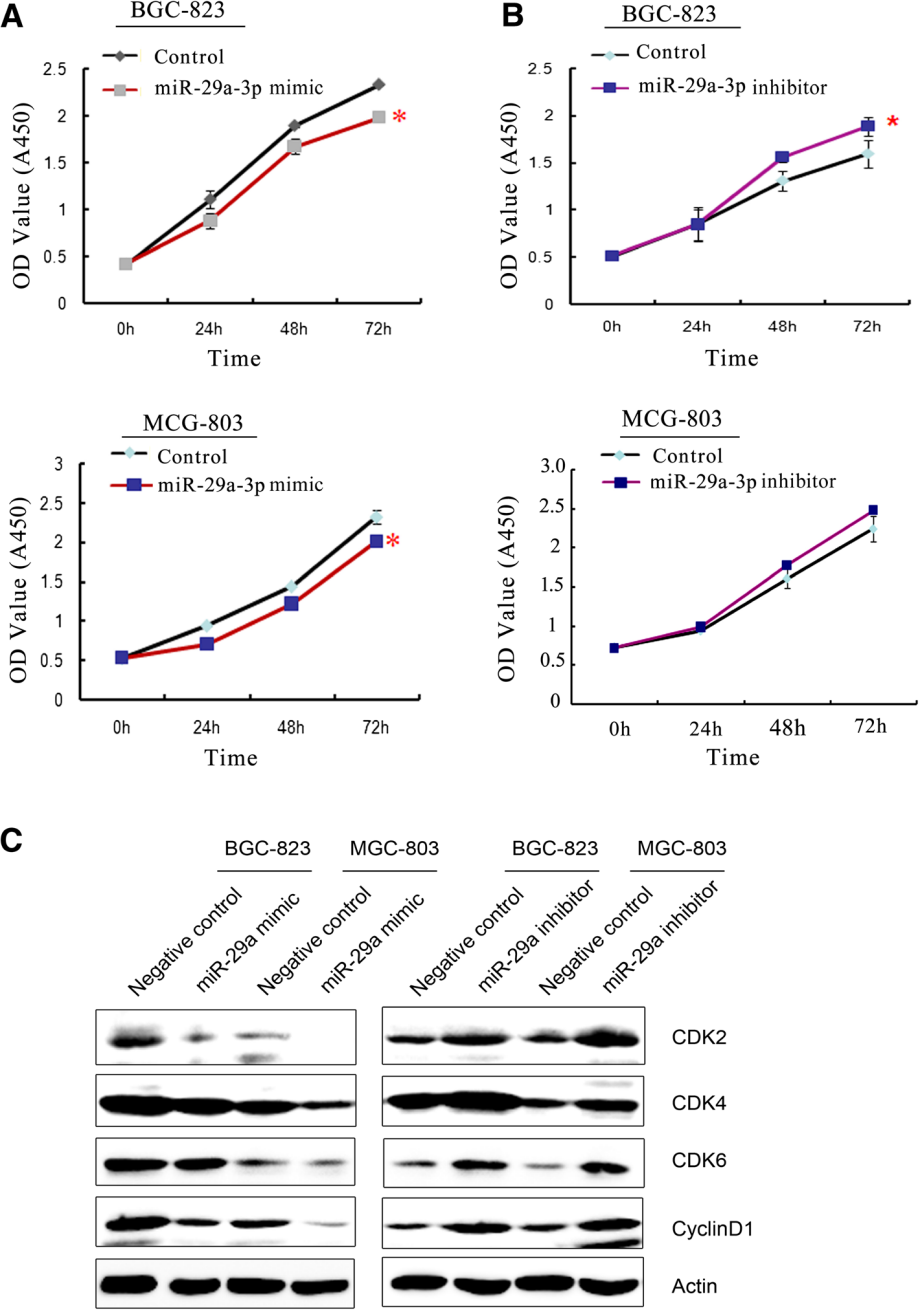
**CCK-8 assay was performed to analyze the effect of miR-29a-3p on cell proliferation ability in GC cells. (A)** The cell growth rates of BGC-miR-29a-3p-mimics (upper) and MCG-miR-29a-3p-mimics (bottom) were detected by the CCK-8 assay. miR-29a-3p significantly decreased cell growth rates (*P < 0.05; independent student’s *t*-test). **(B)** The cell growth rates of BGC-miR-29a-3p-inhibitor (upper) and MCG-miR-29a-inhibitor (bottom) were detected by the CCK assay. miR-29a-3p knockdown significantly increased cell growth rates (*P < 0.05; independent student’s *t*-test). **(C)** Expression of CDK2, CDK4, CDK6, and CyclinD1 were detected in BGC-823 or MCG-803 transfected with miR-29a-3p mimics (left)/inhibitor (right). *β*-actin was used as a loading control. OD, optical density.

miR-29a-3p expression level was correlated with the degree of differentiation which partly depended on cell proliferation. Therefore, we assessed the role of miR-29a-3p by gain- and loss-function experiments on cell proliferation in GC cell lines. BGC-823 and MCG803 cells, which showed moderate miR-29a-3p mRNA, were used to explore the cell proliferation suppression role of miR-29a-3p (Figure 2A,B). BGC-823 and MCG803 cells were transfected with a mimic or inhibitor and their corresponding scramble sequence control, respectively. The cell proliferation suppression effects of miR-29a-3p were evaluated by CCK-8 assay. Cell growth curve assay revealed that cells transfected with miR-29a-3p inhibitor grew more rapidly than the control group, while miR-29a-3p mimics inhibited the cell growth compared to the control. In order to explain the effect of miR-29a-3p on cell proliferation, we further examined the expression of several cell-cycle regulatory proteins in transfected miR-29a-3p mimic or inhibitor cells. The results showed that the expression level of CyclinD1, CDK2, CDK4, and CDK6 were negatively related to the expression level of miR-29a-3p (Figure 2C). These results implied that miR-29a-3p might contribute to the stimulation of cell-cycle progression by enhancing the G1/S transition.

### miR-29a-3p suppressed GC cell migration and invasion

**Figure 3:**
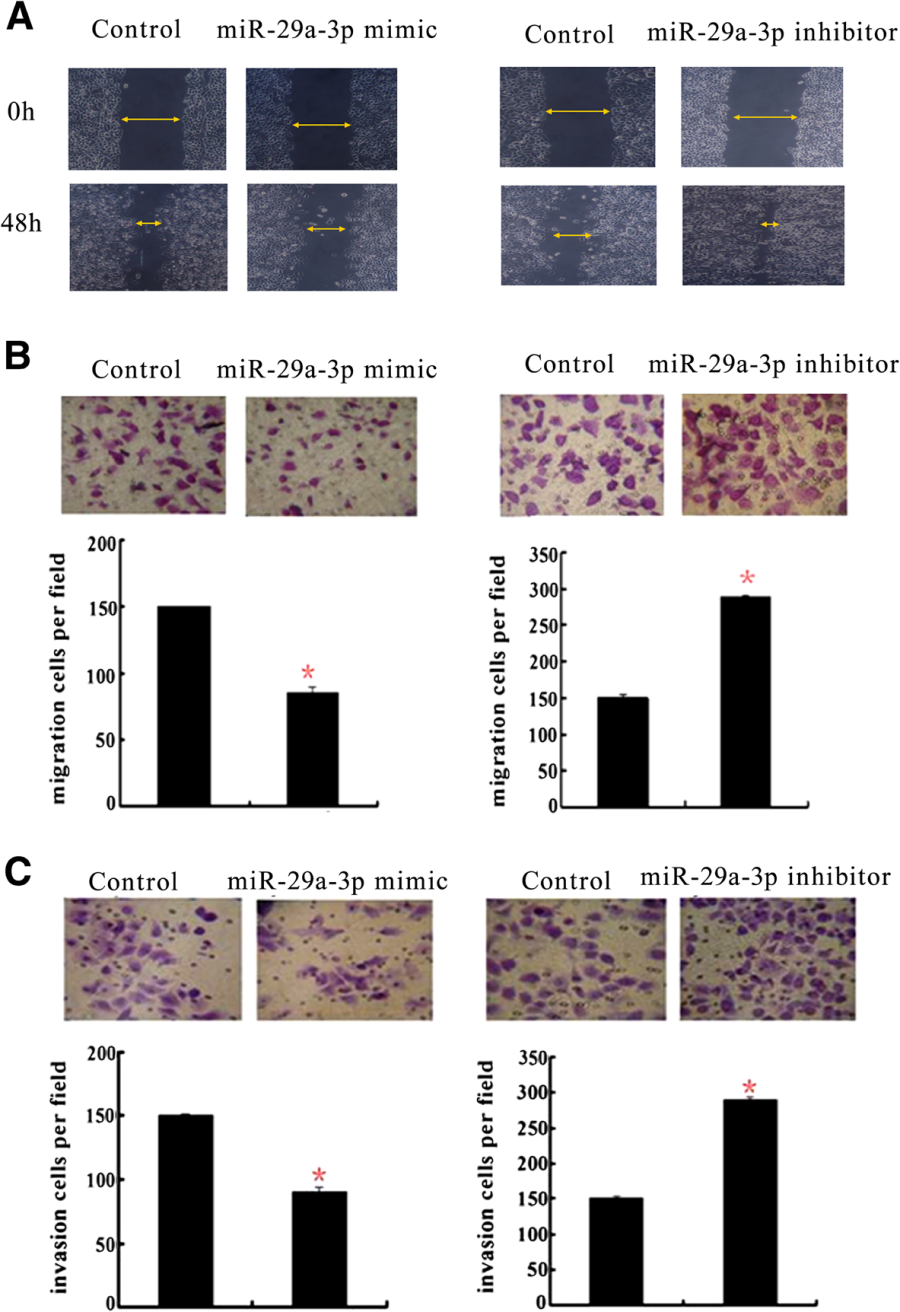
**The effect of miR-29a-3p on GC cell migration and invasion. (A)** Wound healing assays on the confluent layers of miR-29a-3p mimics-transfected BGC-823 cells (left) and miR-29a-3p inhibitor-transfected BCG-823 cells (right). Representative images of wound healing assays were acquired at 0 and 48 h after wounding. Representative images (upper) and bar graphs (bottom) depicting the migration **(B)** and invasion **(C)** ability of BGC-823 cell after the 48 h transfection of negative control mimics or miR-29a-3p mimics (left) (*P < 0.05) and miR-29a-3p inhibitor (right) compared to its control (*P < 0.05).

To investigate the role of miR-29a-3p in GC migration and invasion, miR-29a-3p mimics/inhibitor and their corresponding scramble sequences were transfected into BGC-823 cells. Transwell assay and wound healing method were performed *in vitro*. The efficiency of the transfection was tested by measuring the mature miRNA levels by qRT-PCR (data not shown). After transiently transfecting miR-29a-3p mimics or a negative control into BGC-823 cells, the wound healing assay showed that the forced expression of miR-29a-3p displayed a notable slower recovery compared with control cells (Figure 3A). Similarly, the Transwell migration assay showed that the over-expression of miR-29a-3p was associated with significantly less migration than the control (*P* < 0.05, Figure 3B, left). The over-expression of miR-29a-3p cells also revealed a significant reduction in invasive ability in a Matrigel invasion assay (*P* < 0.05, Figure 3C, left). These results suggested that miR-29a-3p was important not only for GC cell invasion but also for cell migration. To further confirm the suppressive effects of miR-29a-3p on GC cell migration and invasion, BGC-823 was transiently transfected with miR-29a-3p inhibitors or a negative control. The deletion of miR-29a-3p significantly increased the migratory and invasive capabilities of the GC cells, as assessed by wound healing (Figure 3B, right) and a Transwell assay (*P* < 0.05, Figure 3C, right). Collectively, these results indicated that miR-29a-3p effectively abolished GC cell migration and invasion, which therefore might contribute to the early stages of the malignant progression of GC.

## Discussion

miRNA is an important regulator of gene expression at the post-transcriptional level and participates in cell development, differentiation, proliferation, cell-cycle control, apoptosis, and metabolism [4]. Increased evidences have implied that miRNAs function as oncogene and tumor-suppressor genes, regulating the expression and function of their related target genes during the biological process of cancer cells. Our previous study showed that there was distinct miRNA profiling between GC tissues and matched non-tumor tissues based on the miRNA microarray analysis (Additional file 4: Table S4). In the present study, miR-29a-3p was selected as a candidate miRNA due to its down-regulation in tumor tissues. miR-29a-3p is one of the members of miR-29 family, which also includes miR-29b and miR-29c. Accumulating evidences have shown that the aberrant expression of miR-29s are prevalent in multiple cancer types and are involved in complex regulatory process by targeting multiple factors associated with several common pathways, indicating its critical role in carcinogenesis and cancer progression [14-18]. miR-29a has two different transcripts, which are miR-29a-3p and miR-29a-5p, respectively. Neither of them has been reported specific aberrant expression in tumors. Recent studies have found that miR-29a is up-regulated in indolent human B cell chronic lymphocytic leukemia (B-CLL) and acute myeloid leukemia (AML) [19,20]. However, some researches reveal that miR-29a is down-regulated in neuroblastoma, sarcomas, and brain tumors [12,21]. In fact, miR-29a could suppress cell proliferation and induce cell-cycle arrest *via* the down-regulation of p42.3 expression [22]. These inconsistent findings indicate that dysregulation of miR-29a in various cancers may be dependent on the cellular microenvironment, especially with regard to detecting different transcripts of miR-29a-3p. In the present study, we analyzed the expression levels of miR-29a-3p transcript in GC tissue samples and GC cells and its primary biological function on GC cells. We found that decreased expression of miR-29a-3p in GC tumor tissues was related to the degree of cell differentiation. Decreased miR-29a-3p expression promoted gastric cancer cell proliferation *via* reducing the expression of cell-cycle regulators including: CDK2, CDK4, and CDK6. In a way, our finding might be consistent with the reporting of Cui Y *et al.* [22].

Many miRNAs have been identified as key players in metastasis and invasion process of tumor. In prostate cancer, miR-29a is considered as a putative tumor-suppressive miRNA, contributing to cell migration and invasion [23]. miR-29 family plays a dominant role in regulating extracellular matrix genes, such as collagens, LAMA2, integrin β, Mmp2, fibrillin, secreted protein, acidic, and Sparc [16,24], consequently contributing to the promotion of cancer cell migration and metastasis. The present findings showed that miR-29a-3p significantly inhibits the cell invasion and migration ability in GC cells. With the help of bioinformatics prediction (Target scan, miRanda, miRWalk, and miRDB), *ITGA6*, *LAMA2*, and *DNMT3A* were identified as direct targets of miR-29a-3p. Thus, the function of miR-29a-3p in metastasis depends on its target regulation. Although we evaluated its potential target gene *ITGA6*, *LAMA2* expression in miR-29a-3p mimic, and inhibitor-transfected cells (Additional file 5: Figure S5), future studies should be required to validate the association between miR-29a-3p and targets. These results indicate that miR-29a-3p may serve as a potential predictor for prognosis of gastric cancer patients.

## Conclusions

In conclusion, the present study reveals that miR-29a-3p is down-regulated in GC, and it may contribute to the occurrence of gastric cancer *via* influencing cell proliferation, migration, and invasion. These findings provide evidence for the clinical value of miR-29a-3p as targets for GC therapy, although the precise regulatory mechanism and specific targets should be explored in the future study.

## Additional files

Additional file 1: Table S1. miR-29a-3p mimics/inhibitor and control sequences. miR-29a-3p mimic and its control sequences were used to enforce the expression of miR-29a-3p in gastric cancer cells. miR-29a-3p inhibitor and its control sequences were used to knock down the expression of miR-29a-3p in gastric cancer cells. Additional file 2: Table S2. Primers sequences for qPCR analysis. These primers were used to analyze expression of potential target genes of miR-29a-3p. Additional file 3:
**Real-time reverse transcriptase quantitative PCR for detecting miRNA expression.** For the detection of miRNA expression, the primers used for stem-loop RT-PCR and qPCR were synthesized and purified by RiboBio. The methods for analyzing the expression level of miRNA. Additional file 4: Table S4. Down-regulated ≥3-fold microRNA profiling in gastric cancer cases. Information showed in this table was down-regulated miRNA in gastric cancer tissues compared with matched from microRNA array analysis. Additional file 5: Figure S5. The target gene ITGA6, LAMA2, and DNMT3A expression in miR-29a-3p mimic and inhibitor-transfected cells analyzed by qPCR. With the help of bioinformatics prediction (Target scan, miRanda, miRWalk, and miRDB), ITGA6, LAMA2, and DNMT3A were identified as direct targets of miR-29a-3p. qPCR analysis showed that the ITGA6, LAMA2, and DNMT3A mRNA level were inhibited after transfected with miRNA-29a-3p mimic, and after being transfected with miR-29a-3p inhibitor, the ITGA6, LAMA2, and DNMT3A mRNA levels were increased.

## Abbreviations

GC: gastric cancer
qRT-PCR: quantitative reverse-transcription polymerase chain reaction
