# Supplementary material for: Reduced miR-29a-3p expression is linked to the cell proliferation and cell migration in gastric cancer
Source: World J Surg Oncol. 2015 Mar 12;13:101. doi: 10.1186/s12957-015-0513-x (PMC4363339; doi:10.1186/s12957-015-0513-x)
Supplement: Additional file 1: Table S1. — miR-29a-3p mimics/inhibitor and control sequences. miR-29a-3p mimic and its control sequences were used to enforce the expression of miR-29a-3p in gastric cancer cells. miR-29a-3p inhibitor and its control sequences were used to knock down the expression of miR-29a-3p in gastric cancer cells. [file 12957_2015_513_MOESM1_ESM.docx]

**Supplementary**

Table S1 miR-29a-3pmimics/inhibitor and control sequences

| miR-29a-3p mimics | F: 5′-UAGCACCAUCUGAAAUCGGUUA-3′ |
| --- | --- |
|  | R: 5′-ACCGAUUUCAGAUGGUGCUAUU-3′ |
| mimics control | F: 5′-UUCUCCGAACGUGUCACGUTT-3′ |
|  | R: 5′-ACGUGACACGUUCGGAGAATT-3′ |
| miR-29a-3p inhibitor | F: 5′-UAACCGAUUUCAGAUGGUGCUA-3′ |
| inhibitor control | R: 5′-CAGUACUUUUGUGUAGUACAA-3′ |
