# Supplementary material for: Reduced miR-29a-3p expression is linked to the cell proliferation and cell migration in gastric cancer
Source: World J Surg Oncol. 2015 Mar 12;13:101. doi: 10.1186/s12957-015-0513-x (PMC4363339; doi:10.1186/s12957-015-0513-x)
Supplement: Additional file 2: Table S2. — Primers sequences for qPCR analysis. These primers were used to analyze expression of potential target genes of miR-29a-3p. [file 12957_2015_513_MOESM2_ESM.docx]

**Supplementary**

Table S2 Primers sequences for qPCR analysis

| ITGA6 | F- 5′-TCAATTGCTGGAAACATGGA-3′ | |
| --- | --- | --- |
|  | | R- 5′-GGCGGAGGTCAATTCTGTTA-3′ |
| *LAMA2* | | F- 5' -AATTTACCTCCGCTCGCTAT-3' |
|  | | R- 5' -CCTCCAATGTACTTTCCACG-3' |

|  |  |
| --- | --- |
