# Supplementary material for: Reduced miR-29a-3p expression is linked to the cell proliferation and cell migration in gastric cancer
Source: World J Surg Oncol. 2015 Mar 12;13:101. doi: 10.1186/s12957-015-0513-x (PMC4363339; doi:10.1186/s12957-015-0513-x)
Supplement: Additional file 3: — Real-time reverse transcriptase quantitative PCR for detecting miRNA expression. For the detection of miRNA expression, the primers used for stem-loop RT-PCR and qPCR were synthesized and purified by RiboBio. The methods for analyzing the expression level of miRNA. [file 12957_2015_513_MOESM3_ESM.docx]

Supplementary information

***Real-time reverse transcriptase quantitative PCR***

Total RNA was extracted from cells and tissues samples with Trizol reagent (*Invitrogen*, Carlsbad, CA). For the detection of miRNA expression, the primers used for stem-loop RT-PCR and Q-PCR were synthesized and purified by RiboBio. The PCR conditions were 95℃ for 30s, followed by 40 cycles of 95℃ for 30s, 60℃ for 30s, 72℃ for 30s. The reactions were monitored using a preheated real-time instrument (ABI step one). The relative expression ratio of miRNA in gastric cancer tissues and cells was quantified by the 2^−△△CT^ method.
