# Supplementary material for: Reduced miR-29a-3p expression is linked to the cell proliferation and cell migration in gastric cancer
Source: World J Surg Oncol. 2015 Mar 12;13:101. doi: 10.1186/s12957-015-0513-x (PMC4363339; doi:10.1186/s12957-015-0513-x)
Supplement: Additional file 4: Table S4. — Down-regulated ≥3-fold microRNA profiling in gastric cancer cases. Information showed in this table was down-regulated miRNA in gastric cancer tissues compared with matched from microRNA array analysis. [file 12957_2015_513_MOESM4_ESM.docx]

**Supplementary**

Table S4 Down-regulated ≥3 fold microRNA profiling in gastric cancer cases

| No. | Gene name | Down fold |
| --- | --- | --- |
|  | hsa-miR-31-5p | 0.116152707 |
|  | hsa-miR-625-5p | 0.214691047 |
|  | hsa-miR-147b | 0.232581967 |
|  | hsa-miR-1252 | 0.062021858 |
|  | hsa-miR-1204 | 0.217004884 |
|  | hsa-miR-1539 | 0.120042306 |
|  | hsa-miR-29a-3p ***** | 0.279530625 |
|  | hsa-miR-125b-5p | 0.173211844 |
|  | hsa-miR-21-3p | 0.27258014 |
|  | hsa-miR-1229 | 0.031359366 |
|  | hsa-miR-369-5p | 0.17814789 |
|  | hsv2-miR-H9-3p | 0.237847974 |
|  | hsa-miR-3169 | 0.174436475 |
|  | hsa-miR-4304 | 0.279098361 |
|  | hsa-miR-532-3p | 0.206739526 |
|  | hsv2-miR-H13 | 0.279098361 |
|  | hsv1-miR-H18 | 0.186065574 |
|  | hsa-miR-1225-3p | 0.261654713 |
|  | hsa-miR-513b | 0.18112578 |
|  | hsa-miR-139-5p | 0.218900675 |
|  | hsa-miR-3692-3p | 0.169150522 |
|  | hsa-miR-548o-3p | 0.125719982 |
|  | hsa-miRPlus-A1031 | 0.232581967 |
|  | hsa-miR-346 | 0.223278689 |
|  | hsa-miR-4252 | 0.155054645 |
|  | hsa-miR-3679-3p | 0.28689525 |
|  | hsa-miR-3945 | 0.232581967 |
|  | hsa-miR-154-3p | 0.143127364 |
|  | hsa-miRPlus-D1100 | 0.13954918 |
|  | hsa-miR-484 | 0.074240628 |
|  | hsa-miR-3117-3p | 0.032080271 |
|  | kshv-miR-K12-4-3p | 0.054725169 |
|  | hsa-miR-661 | 0.124043716 |
|  | hsa-miR-105-3p | 0.04228763 |
|  | hsa-miR-1237 | 0.290727459 |
|  | hsa-miR-129-2-3p | 0.295685602 |
|  | hsa-miR-2355-5p | 0.245423916 |
|  | hsa-miR-1273a | 0.248087432 |
|  | hcmv-miR-US33-5p | 0.069427453 |
|  | hsa-miR-495 | 0.229003783 |
|  | hsa-miR-1301 | 0.286885246 |
|  | hsa-miR-138-2-3p | 0.071563682 |
|  | hsa-miR-671-5p | 0.181527389 |
|  | hsa-miR-3176 | 0.044301327 |
|  | hsa-miR-4269 | 0.203786105 |
|  | hsa-miR-1181 | 0.223278689 |
|  | hsa-miR-211-5p | 0.186065574 |
|  | hsa-miR-628-3p | 0.152966737 |
|  | hsa-miR-3938 | 0.197342275 |
|  | hsa-miR-101-5p | 0.232581967 |
|  | hsa-miR-29c-3p | 0.23580028 |
|  | hsa-miR-127-5p | 0.293787748 |
|  | hsa-let-7f-5p | 0.169150522 |
|  | kshv-miR-K12-6-5p | 0.253725782 |
|  | hsa-miR-3940-3p | 0.109450338 |
|  | hcmv-miR-US5-1 | 0.169150522 |
|  | hsv2-miR-H9-5p | 0.155054645 |
|  | hsa-miR-3137 | 0.265807963 |
|  | hsa-miR-642a-5p | 0.295200189 |
|  | hsa-miR-760 | 0.265807963 |
|  | hsa-miR-145-5p | 0.275839966 |
|  | bkv-miR-B1-5p | 0.257801699 |
|  | hsa-miR-1323 | 0.206739526 |
|  | hsa-miR-338-3p | 0.253725782 |
|  | hsv1-miR-H15 | 0.189511233 |
|  | hsa-miR-520b/hsa-miR-520c-3p | 0.132903981 |

* candidate gene for the present study
