# Supplementary figures and images for: Reduced miR-29a-3p expression is linked to the cell proliferation and cell migration in gastric cancer
Source: World J Surg Oncol. 2015 Mar 12;13:101. doi: 10.1186/s12957-015-0513-x (PMC4363339; doi:10.1186/s12957-015-0513-x)

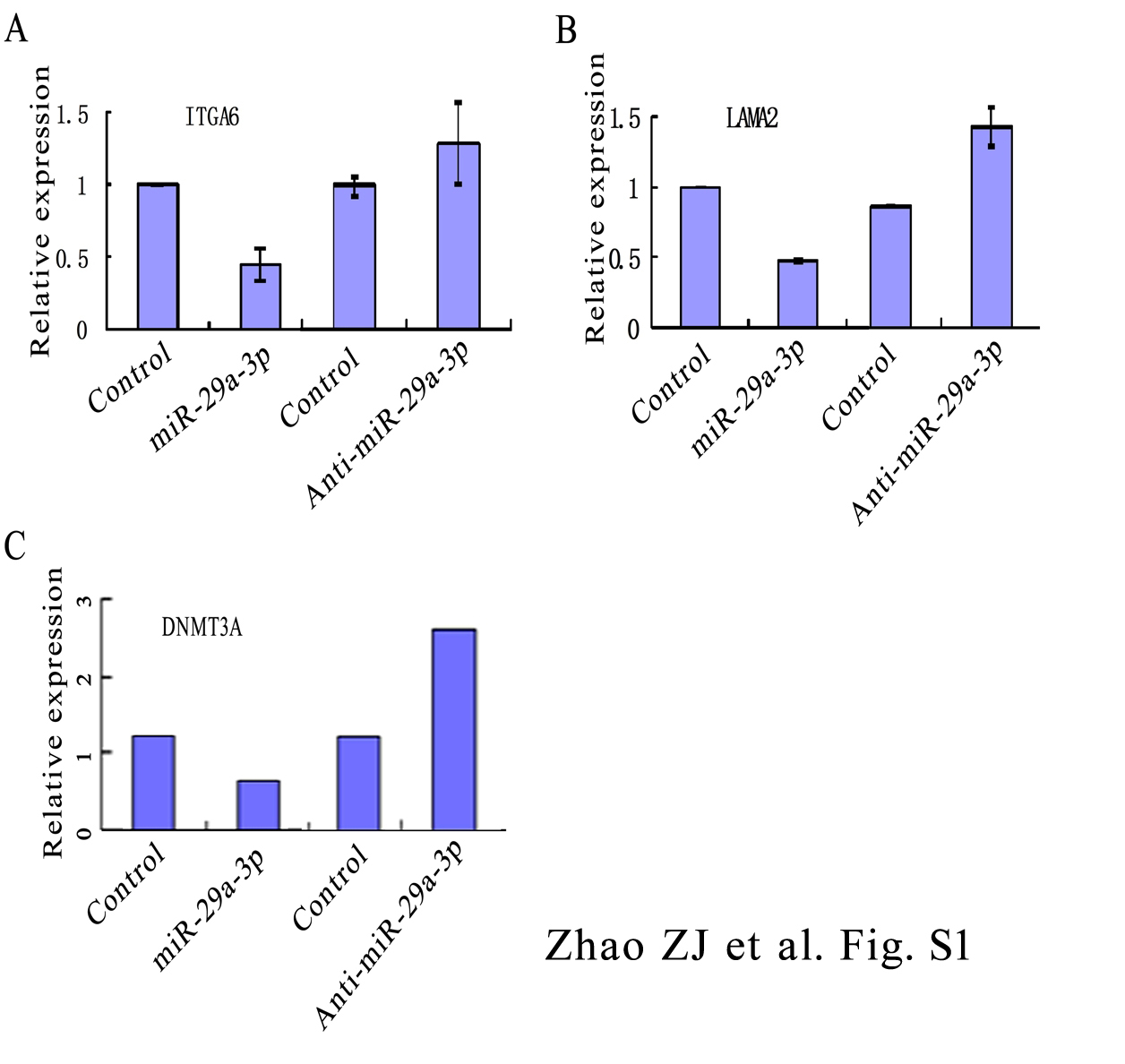

Supplement: Additional file 5: Figure S5. — The target gene ITGA6, LAMA2, and DNMT3A expression in miR-29a-3p mimic and inhibitor-transfected cells analyzed by qPCR. With the help of bioinformatics prediction (Target scan, miRanda, miRWalk, and miRDB), ITGA6, LAMA2, and DNMT3A were identified as direct targets of miR-29a-3p. qPCR analysis showed that the ITGA6, LAMA2, and DNMT3A mRNA level were inhibited after transfected with miRNA-29a-3p mimic, and after being transfected with miR-29a-3p inhibitor, the ITGA6, LAMA2, and DNMT3A mRNA levels were increased. [file 12957_2015_513_MOESM5_ESM.jpeg]
